# Medicalization of global health 2: the medicalization of global mental health

**DOI:** 10.3402/gha.v7.24000

**Published:** 2014-05-16

**Authors:** Jocalyn Clark

**Affiliations:** 1 icddr,b, Dhaka, Bangladesh; 2 Department of Medicine, University of Toronto, Toronto, Canada

**Keywords:** global health, mental health, medicalization, sociology

## Abstract

Once an orphan field, ‘global mental health’ now has wide acknowledgement and prominence on the global health agenda. Increased recognition draws needed attention to individual suffering and the population impacts, but medicalizing global mental health produces a narrow view of the problems and solutions. Early framing by advocates of the global mental health problem emphasised biological disease, linked psychiatry with neurology, and reinforced categories of mental health disorders. Universality of biomedical concepts across culture is assumed in the globalisation of mental health but is strongly disputed by transcultural psychiatrists and anthropologists. Global mental health movement priorities take an individualised view, emphasising treatment and scale-up and neglecting social and structural determinants of health. To meet international targets and address the problem's broad social and cultural dimensions, the global mental health movement and advocates must develop more comprehensive strategies and include more diverse perspectives.

Growing recognition of the breadth of mental health issues around the world, now said to comprise up to 13% of the total disease burden, has led mental health to be considered a major issue of global concern, especially since over 70% of the burden lies in low- and middle-income countries (LMICs) (1). The reported ‘treatment gap’ reinforces this vast inequity: while worldwide at least two-thirds of all persons with mental illnesses go untreated, in low-resource countries this figure is said to exceed 90% (2). Advocates call it a ‘global health scandal’ and a ‘crisis’ (2, 3).

Since the launch of an organised movement for mental health to coincide with a high-profile 2007 series in *The Lancet* (4), numerous journal articles, special series, and reports have positioned mental health as an issue facing many individuals and countries around the world, reinforcing it as much more than a ‘rich country problem’. Major support from the US National Institutes of Mental Health, the UK's Department for International Development (DFID), and Grand Challenges Canada has further elevated the profile and recognition of mental health issues. Advocacy appears to draw strength from the sort of orphan status of mental health within the global health field and its relative neglect on the agenda to date: mental health was essentially ignored in the Millennium Development Goal programme and failed to elevate to prominence at the 2011 United Nations special assembly on non-communicable disease (5, 6). Still, mental health gained the backing of WHO with two world health reports and its 2011 Mental Health Gap Action Programme (mhGap) to extend coverage of services for mental disorders in the developing world, and in late 2013 the comprehensive mental health action plan was endorsed, a formal recognition of the importance of mental health by WHO's member states.

This new field of ‘global mental health’ has spawned research units, training centres, journals, and the influential Movement for Global Mental Health (MGMH), an international coalition that has grown to over 3,000 organisations and individuals in 60 countries. Its main focus is the treatment gap, and aims to scale-up health services and treatment coverage for mental disorders in all countries, especially in LMICs, recognising the importance of human rights protection and scientific research (7).

Viewed through the lens of medicalization (Box 1), global mental health is a fascinating phenomenon. As I have argued elsewhere (5), there exists a paradox, which is growing as mental health is globalising: on one hand, badly needed attention to mental health issues and people's suffering is welcomed and essential, but on the other hand the predominance of medical framings of global mental health has created conditions for disease mongering and medicalization. That the global mental health agenda may be medicalized is apparent in much of its early advocacy and priorities. Building on other critiques from commentators in the fields of transcultural psychiatry and social sciences, I use a medicalization lens – described in-depth in Paper 1 of this series (8) – to critique how the problem of and solutions to mental health issues around the world are framed, and to uncover areas where more diverse perspectives and strategies may be needed to address the problem's broad social and cultural dimensions.

***Box 1.*** Medicalization lens

Medicalization is a process by which human problems come to be defined and treated as medical problems. It involves the application of a biomedical model that sees health as freedom from disease, and is characterised by reductionism, individualism, and a bias towards the technological. Medicalization analyses have roots in social constructionism and sociology, revealing the ways that such varied conditions as addiction, childbirth, infant feeding, sadness, erectile dysfunction, and death have come to be medical issues to be treated (8).

Medicalization is understood to be expanding rather than contracting in modern life, and while it can be sought or resisted by patients, doctors, or other actors in the health field, it is considered largely harmful and costly to individuals and societies: pathologising normal behaviour, disempowering individuals, decontextualising experience, and depoliticising social problems. As Parens argues (9): *Insofar as medicine focuses on changing individuals’ bodies to reduce suffering, its increasing influence steals attention and resources away from changing the social structures and expectations that can produce such suffering in the first place*.

Medicalization can play out at the level of interaction or of definition and agenda-setting, and analyses of medicalization are particularly valuable at the definitional level. As outlined in-depth in Paper 1 of this series (8), a medicalization lens can be useful for critically examining the contemporary global health agenda, exploring how and why certain issues elevate to prominence and raising questions such as:

- How is the issue framed as a problem in global health and what solutions are presented?
- To what extent and how are priority issues defined as diseases, strategies for treatment and other health care technologies recommended, and individual versus social solutions advanced?
- What is the role of medical providers or of a biomedical model conceived to be or promoted?
- How do advocacy groups, civil society, and/or industry reinforce, benefit, or challenge the medicalization of the global health issue?
- Who benefits from the framing of the issue and its solutions, and what is discounted?

A medicalization lens helps uncover areas where the global health agenda and its framing of problems are shifted towards medical and technical solutions, neglecting necessary social, community, or political action.

Cases examined in this series on the Medicalization of Global Health (8) include: the global mental health movement (this paper), the non-communicable diseases agenda (10), and the universal health coverage campaign (11).

I trace three features.

## Real disease

First, a feature in the success of global mental health's raised profile has been attempts by advocates to emphasise the biological basis of mental health problems, and further, to link these to disorders of the brain. In one way, this has likely served to destigmatise patients and locate their troubles in ‘real’ pathology of the brain as distinct from ‘psychiatric disturbances arising from weak moral fibre or bad breeding’ (12). For example, an advocate described the scenario where once, Alzheimer disease was simply ‘“growing old badly” but is now confirmed as a disease entity through advances in biology and neuroscience’ (13). In another way, this has collected conditions with less biological confirmation together with established neurological *disease*. Indeed, while the movement is for global mental *health*, the coalition defines their priorities as including a range of mental, neurological, and substance use (MNS) *disorders*, reinforcing a set of disease categories to frame the problem under a single banner. Both WHO and the Grand Challenges in Global Mental Health initiative adopt this framing of mental health: ‘all conditions that affect the nervous system and are leading causes of disease burden: depression, anxiety disorders, schizophrenia, bipolar disorders, alcohol and drug use disorders, mental disorders of childhood, migraines, dementias, and epilepsy – collectively identified as mental, neurological, and substance use (MNS) disorders’ (14). Essentially, similarly to the Institute of Medicine definition of global health (15), proponents of global mental health have contributed to medicalization of the field by identifying priority areas that are diseases classified medically.

The framing of this new constellation of disorders as having a potentially shared biological base – where there might be ‘power in numbers’ – may be a boon for a burgeoning field and offers the possibility of greater physiological and chemical understanding and future cures (16) but also reinforces global mental health as a medical kind of problem.

In fact, there is a great deal of dispute about the evidence for the biological base of some of the most promoted conditions – depression and post-traumatic stress disorder in particular (16) – but advocacy nevertheless has emphasised the conditions as disease entities in need of treatment. Clearly, prioritising the diagnosis and treatment of some mental health conditions – severe disorders such as schizophrenia where sufferers may be subject to extreme abuse and the evidence for treatment is strong – draws on a more robust case than less clear areas such as depression, intellectual disability, or ‘adjustment disorders’. Nevertheless, the tying of mental health conditions to neurology served to also, at least in part according to Hyman, validate not just the experiences of individuals suffering but to dignify the profession of psychiatry, putting them on the same level as cardiologists (17). This then allows the global mental health field to align with a ‘more medical’ specialty, gaining credibility. The medicalizing consequences of promoting disorders and disease and an elevated role for physicians is reinforced by the dominance of the professional view in the global mental health movement and discourse, whose agendas are advanced while others are relatively disqualified and subjugated. While ‘end-users’ or patients, other members of civil society, community-based groups such as the innovative BasicNeeds organisation, and social scientists are represented among the MGMH and other initiatives, the most visible proponents of global mental health tend to be clinicians and others professionals working within biomedical disciplines, as others have argued (18).

## Universality

Second, there is another use of shared biology in the framing of global mental health that reinforces the medicalized view: the argument that shared biology unifies experiences across culture. The very notion of a global mental health (and its priorities to scale-up Western biomedical treatment) relies upon the idea that a universal set of concepts, causes, symptoms, and experiences can be applied across regions and cultures of the world. That a range of conditions is included in the global mental health stable reinforces an assumption of universality for all. But this idea has been strongly challenged, especially from transcultural psychiatrists who question how globally valid the concepts and assumptions guiding the global mental health agenda are. The movement's most ardent critic, Summerfield, for example, calls into question the very existence of the vast burden reported by advocates, especially in areas of depression, anxiety, and post-traumatic stress disorder, and argues that this is revealed only when Western psychiatric categories and measures are applied (19–21). That disease categories are socially and historically constructed is shown by the fact that homosexuality was a classified mental illness until 1980 (21) and ‘Internet use gaming disorder’ only since 2013 (5).

Anthropologists, too, contrast the dominant view of global mental health (where evidence from high-income countries about medical interventions is said to be exported and adapted to LMICs in a top-down manner) with a cultural view that emphasises local priorities, resources, and solutions (18). Understanding mental health requires not just acknowledgment of the physical realms emphasised by biomedicine, say anthropologists, but also the moral, spiritual, emotional, and social (22). Cross-cultural evidence shows how depression in the medicalized fashioning may in others be ‘thinking too much’, ‘fall of heart or mind’, or sadness, or depending on the social circumstances may be distress due to displacement, conflict, injustice, or social suffering (19, 21). Worse, exporting a biomedical model may exacerbate problems: ‘Offering the latest Western mental-health theories, treatments and categories in an attempt to ameliorate the psychological stress sparked by modernization and globalization is not a solution; it may be part of the problem. When we undermine local conceptions of the self and modes of healing, we may be speeding along the disorienting changes that are at the very heart of much of the world's mental distress’ (23).

## Individualistic solutions

The third feature involves taking an individual view of solutions. The priorities advanced for alleviating the problems of global mental health – favouring health care treatment and technologies – also serve to position it as a medical problem. While there is recognition on the part of GMH advocates of the social *drivers* of poor mental health – poverty, social inequalities, injustice – and that the human rights of those suffering must be promoted and protected (2, 24, 25), this stands at odds with the main focus on scaling-up of health care services – that is, medical treatments including medication targeted towards individuals (26). As Campbell and Burgess have argued, ‘the [global mental health] discipline's literature concedes the social and economic determinants of poor mental health, but the thrust is of the global deployment of Western biomedical models of mental disorder’ (18). They show how notions of community involvement and participation are viewed through a narrow frame of the medical model of disease and recovery (18); communities appear to be defined as patients and health workers charged with helping in the scaling-up of medically oriented mental health services (27). Community participation can reduce to a mere ‘magic bullet’ if not properly accompanied by wider efforts to create contexts that support effective grassroots mobilisation, argues Petersen (28), who shows in South Africa how community involvement in mental health care delivery improved participation and awareness of local health services but failed to adequately address gender inequities, injustices, and poverty that were the underlying causes. Therefore, even attempts to introduce and emphasise psychosocial interventions in addition to pharmacological interventions, as the mhGap does (http://www.who.int/mental_health/mhgap/en/), can reinforce individualism.

The individualistic approach of the global mental health agenda that gives prominence to scaling-up of medical treatments carries other implications. That such priorities create vast profit-making opportunities for pharmaceutical companies is evident in the phenomenon of disease mongering, demonstrated mostly in North America, which ‘creates’ diseases and patients and has been shown to be particularly significant in mental health (5, 29). As others argue, over-diagnosis risks unnecessary tests and treatment, the stigma associated with being labelled mentally ill, the considerable costs of testing and treatment, and wasting resources that could be better used elsewhere (5, 29, 30). With regard to antidepressants and psychotropics specifically, the evidence base for their effectiveness is incomplete, flawed, contestable, or falsified; they can have harmful side effects; and patient populations from LMICs were not sufficiently included in clinical trials (16, 31, 32). And with the mental health field globalising, the risks and consequences of overdiagnosis and overtreatment may become more than just a problem of high-income countries, especially if and when new treatment areas such as bipolar disorder and psychosis are advocated in LMICs. One market research firm says the global mental health pharmaceutical market will grow to US$88.3 billion by 2015 (33), and at the 2013 World Health Assembly, the International Federation of Pharmaceutical Manufacturers’ Association highly commended WHO for its leadership and affirmed the pharmaceutical industry's active engagement in the ‘fight against mental and neurological disorders’ with over 200 compounds said to be in research and development, and several on the ground already (34). Growth of pharmaceutical markets depends upon continued growth in the number of diagnosed mental health patients; therefore, it comes as no surprise that pharmaceutical industry approaches support a medicalized framing of global mental health.

Anthropological accounts confirm the inadequacies of this medicalized approach, where simply increasing access to antidepressant or psychotropic medications can result in adverse effects that disrupt the ability to work (35), create dependencies in the case of benzodiazepines, eclipse the use of group therapy or other non-pharmaceutical treatments, and contribute to family conflict (36). These medicalized approaches or ‘packages of care’ in Han's case study (36) were often presented as modern treatments for mental health but are detrimentally disconnected from the ‘life worlds’ of individuals. Still, as Patel and colleagues’ research in India suggested, medication treatment may be more acceptable to individuals with common mental health issues than non-pharmaceutical approaches such as talking therapies, especially in a group environment (37).

The most vocal critics of the global mental health movement have suggested that the field is driven by psychiatry and the pharmaceutical industry (13). But even more generous views would see that any further focus on treatment or biomedicine alone will not meet the established goals of the WHO comprehensive action plan to 2020, nor the vision for the place of global mental health in the post-2015 development agenda posted recently by the MGMH that appears to acknowledge the need for a vastly broader vision of human rights protection, prevention of discrimination, and the provision of quality, culturally appropriate care (38).

## How can the medicalization of global mental health be challenged?

The medical framing of global mental health is apparent and influential but can be challenged.

More extensively drawing on the views of those most vulnerable will help counter the dominant medical professional view in agenda-setting and may provide an alternate framing to the causes and consequences of mental health issues. Patient views may reveal the positive and beneficial aspects of medicalization. A medical diagnosis or label, for example, can validate an individual's experiences or serve as legitimation of their suffering or poor health when it comes to disability or legal claims, and thus may be strategically sought by patients. Nevertheless, it must be acknowledged that medical labels can be double-edged: a PTSD diagnosis in refugee camps in Somalia, for example, was said to increase access to health care but in some cases was a barrier to refugees labelled mentally ill who were then denied asylum to certain countries (13). In addition, patient views, if not integrated with broader contexts, can also reinforce individualistic solutions, such as when patient groups lobby for access to new or experimental drugs.

More of the patient perspective is clearly needed, but to expand a medicalized view it would seem vitally important to better incorporate local meanings and norms and other aspects of culture that influence global mental health, as emphasised by anthropologists. Even the notion of community has different meaning in the medicalized and the local cultural contexts, and these areas of disagreement must be explored rather than recast in authoritative medical or technical concepts. Significant efforts such as PRIME (39), the 6-year research programme funded by DFID to generate evidence on implementation and scale-up of mental health care in LMICs, has explicitly stated that it aims to integrate facility and community-based care, and could benefit from expanded notions of community and culture. The process of uncovering these could serve as the opportunity for more social scientific perspectives and a broader civil society to be engaged in the global mental health movement.

Expanded use of participatory research methodologies in the global mental health area would seem particularly apt. Described as seeking to understand and improve the world by changing it, and combining social scientific perspectives with community involvement, participatory action research aims to ‘systematize local experience and organize shared collective analysis on relationships and causes of problems’ (40). It can provide a challenge and an alternative framing of issues to those generated by normative analytic frameworks in global health research (biostatistics, epidemiology, health economics), helping expand and politicise the agenda, as appears to be called for.

Indeed, the human rights work in global mental health seems particularly weak compared to the treatment scale-up and research aims of the main movement. Strengthening the human rights aspect is essential to address the poverty and the inequities that are at the core of the global mental health problem, and cannot be achieved by health care or biomedical interventions alone (22, 25). Instead, multi-sector action is needed to improve income distribution, social development, education, and gender equity, all of which are relatively absent in the current framing of global mental health. Again, participatory research can help here. But this does not absolve the health sector at the centre of the global mental health agenda-setting: health workers can use their influence to advocate for genuine and substantive promotion and protection of the basic needs and human rights of people with mental illness (and not merely a narrow view of human rights to do with access to health care), and this requires recognition and incorporation of social justice frameworks and political aims. Emerging critical medical organisations such as the international critical psychiatry network are concerned with these broader contexts, in particular through raising awareness of the overreliance on diagnostic classifications and psychopharmacology (see http://www.criticalpsychiatry.co.uk/). It also requires an expansion of the individualistic approach that has dominated the global mental health agenda to date, which fails to adequately position those with mental health issues in the contexts of their daily lives, work, families, communities, religion, and the economic system (18, 22, 24, 35).

In fact, an unfortunate consequence of the nature versus culture debate in relation to the global mental health movement is that the social, political, and economic determinants of health get obscured, which may be why they seem missing in the predominant agenda and research priorities.

Work to broaden definitions of global mental health will be no small feat. A key challenge is to address the need – when advancing the agenda and bringing profile and resources to the problem of global mental health – to focus on standards, agreements, feasibility, affordability, and measurable results, which is what policymakers need and want, while at the same time broadening the definitions, solutions, and strategies to better account for the full dimensions of what it will take to achieve global mental health.
